# Intraspinal neuroblastoma: Treatment options and neurological outcome of spinal cord compression

**DOI:** 10.3892/ol.2014.2795

**Published:** 2014-12-12

**Authors:** MOHAMED FAWZY, MOHAMED EL-BELTAGY, MAGED EL SHAFEI, MOHAMED SAAD ZAGHLOUL, NAGLAA AL KINAAI, AMAL REFAAT, SARAH AZMY

**Affiliations:** 1 Pediatric Oncology Department, National Cancer Institute, Cairo University, Cairo, Egypt; 2 Department of Pediatric Oncology, Children’s Cancer Hospital of Egypt (CCHE/57357), Cairo, Egypt; 3 Department of Neurosurgery, Kasr El-Aini Faculty of Medicine, Cairo, Egypt; 4 Department of Neurosurgery, Children’s Cancer Hospital of Egypt (CCHE/57357), Cairo, Egypt; 5 Department of Surgical Oncology, National Cancer Institute, Cairo University, Cairo, Egypt; 6 Department of Surgical Oncology, Children’s Cancer Hospital of Egypt (CCHE/57357), Cairo, Egypt; 7 Department of Radiotherapy, National Cancer Institute, Cairo University, Cairo, Egypt; 8 Department of Radiotherapy, Children’s Cancer Hospital of Egypt (CCHE/57357), Cairo, Egypt; 9 Department of Pathology, National Cancer Institute, Cairo University, Cairo, Egypt; 10 Department of Pathology, Children’s Cancer Hospital of Egypt (CCHE/57357), Cairo, Egypt; 11 Department of Radiology, National Cancer Institute, Cairo University, Cairo, Egypt; 12 Department of Radiology, Children’s Cancer Hospital of Egypt (CCHE/57357), Cairo, Egypt; 13 Department of Research, Children’s Cancer Hospital of Egypt (CCHE/57357), Cairo, Egypt

**Keywords:** neuroblastoma, intraspinal, cord compression, treatment, outcome

## Abstract

Malignant spinal cord compression (MSCC) is a common complication of cancer. Paraspinal neuroblastoma (NB) in the thoracic, abdominal and pelvic regions may extend into the neural foramina causing compression of nerve roots and even the spinal cord. The prompt initiation of specific treatment can improve the neurological outcome. The aim of the present study was to review the clinical features, the management received and the factors that may affect the outcome of patients with MSCC caused by paraspinal NB. During a period between July 2007 and December 2012, a total of 576 NB patients were treated at the Children’s Cancer Hospital (Cairo, Egypt). Intraspinal disease extension was present in 51 patients (9%). The children with intraspinal disease extension were reviewed for disease pattern, neurological manifestations and treatment outcome. Children with intraspinal disease extension had an equal male to female ratio (1:1), and approximately two-thirds of patients (34/51) had a clinically manifested cord compression. The duration of neurological manifestations was >4 weeks in 58.8% (20/34) of symptomatic patients and ≤4 weeks in 41.2% (14/34). Subsequent to starting treatment, neurological manifestations showed a complete recovery in 16 patients (47.1%), partial in 11 (32.4%), and stationary course was found in 7 (20.6%). Manifestations of ≤4 weeks in duration carried an improved outcome compared with longer time compression, with a complete recovery in 78.6%, versus 25% for patients with a longer symptom duration (P=0.008). The upfront treatment, patient age and site of the primary tumor did not significantly affect the neurological outcome. Spinal cord compression in NB can be effectively managed with upfront chemotherapy. Initial surgical decompression should be reserved for benign variants only, including ganglioneuroma. Neurological manifestations of <4 weeks duration upon presentation are usually reversible.

## Introduction

Malignant spinal cord compression (MSCC) is a serious, common complication of cancer (1). The most common extracranial solid tumor in childhood is neuroblastoma (NB) and this is the most frequently diagnosed neoplasm during infancy as well. In 65% of cases, primary tumors occur within the abdomen and at least half of these arise in the adrenal medulla. Other common sites of disease include the neck, chest and pelvis (2). Paraspinal tumors in the thoracic, abdominal and pelvic regions may extend into the neural foramina causing compression of nerve roots and the spinal cord (3). Neurological signs and symptoms due to cord compression by a tumor is one of the main emergencies that can occur in pediatric oncology (4). However, numerous studies indicate that the majority of these patients that present with MSCC are diagnosed only when they become unable to walk. Despite the widespread availability of good diagnostic technology, magnetic resonance imaging (MRI) remains the instrument of choice for documenting the involvement of the inter-vertebral foramina (1,3). Although management of patients has not been widely consistent, undoubtedly prompt initiation of specific treatment can improve the neurological outcome (3). The aim of the present study was to review the clinical features, management and factors predicting the clinical outcome in MSCC caused by paraspinal NB in a group of children treated at the Children’s Cancer Hospital of Egypt (CCHE; Hospital 57357; Cairo, Egypt).

## Patients and methods

### Patients

A total of 576 NB patients, diagnosed in the period between July 2007 and June 2012 at CCHE, were retrospectively reviewed for disease pattern, intraspinal extension and neurological manifestations (symptoms/signs). In total, 51 out of these 576 patients had clinical and/or radiological evidence of MSCC. The clinical outcome for these patients was thoroughly studied.

### Diagnosis and treatment

Diagnostics, staging and risk categorization of the study patients included physical examination, histopathological classification and imaging studies [computed tomography, MRI and radioisotopic bone and MIBG (meta-iodo-benzyl-guanidine) scans] at initial presentation. The presence of marrow disease was determined by bilateral bone marrow aspirate and biopsy. Patient history, medical records, radiographs and surgical studies were comprehensively reviewed to determine the extent of intraspinal involvement, presence of neurological manifestations and the type, severity and duration of such manifestations. Neurosurgical decompression was performed if the tumor had a benign histology or if neurological manifestations deteriorated within the 48–72 h following the initiation of chemotherapy. Only four patients underwent primary surgical decompression in the form of posterior laminectomy and excision of the epidural section of the tumor to relieve any compression on the cord for tumors histologically diagnosed as ganglioneuroma based on true-cut biopsies. Of these four patients, three were found to have ganglioneuroblastoma post-operatively. All other patients who did not fit either of the two conditions indicated for local intervention were administered etoposide (100 mg/m^2^ d1–3) and carboplatin (560 mg/m^2^ d1) until completion of their disease stratification workup, and then continued on the corresponding risk protocol. Dexamethasone was initiated for 1–2 days prior to chemotherapy at a dose of 16 mg/m^2^ while awaiting definite pathological diagnosis in 15 of the study patients. The neurological status was clinically re-assessed within 48–72 h of initiating treatment in addition to further MRI studies, particularly for patients showing no or unsatisfactory early clinical recovery of their neurological manifestations. Clinical evaluation of the neurological outcome was defined as follows: Stage 1, complete recovery (fully regained motor power and skills, reflexes and no residual sensory or any other neurological symptoms or signs); stage 2, stationary (no recovery) with absent signs of neurological improvement and persistent symptoms/signs of the same intensity; stage 3, partial recovery (between stages 1 and 2); and stage 4, progressive course (newly developed neurological symptoms/signs or worsening of previously present manifestations).

### Statistical methods

All data were analyzed using SPSS 16.0 software (SPSS, Inc., Chicago, IL, USA). The association between the duration and outcome of neurological manifestations and various study parameters were analyzed using the χ^2^ test. The factors known to be associated with the prognosis were tested by univariate analysis. P<0.05 was considered to indicate a statistically significant difference.

## Results

Of the 576 NB patients reviewed, intraspinal disease extension was radiologically confirmed in 51 (9%), whereas clinically manifested spinal cord compression at the time of diagnosis was found in 34 patients representing 6% of the total patients reviewed. Clinical characteristics, pathology subtypes and the biological data of the 51 patients with intraspinal extension are shown in Table I.

The 34 study patients with neurological deficits at presentation were followed up for 6.2–51.7 months (median, 14.8 months). Males and females were equally represented (17 each) with a median age of 31.8 months (range, 2.2–159.6 months). Advanced International NB Staging System disease stages 3 and 4 were found in 88% of cases, while high-, intermediate- and low-risk disease was identified in 65, 29 and 6% of patients, respectively. Neurological manifestations were present for >4 weeks duration in 58.8% (20/34) of symptomatic patients. By contrast, 41.2% (14/34) of patients had neurological manifestations for a duration of ≤4 weeks. Subsequent to receiving upfront treatment [chemotherapy only (n=16), concomitant steroids with chemotherapy (n=14) and spinal laminectomy (n=4)], 16 patients (47.1%) showed a complete recovery of their neurological manifestations (returned to normal), 11 patients (32.4%) exhibited partial (less than complete) recovery and the remaining seven patients (20.6%) exhibited a stationary course (no improvement) of their neurological symptoms and signs (Table II).

An improved outcome was significantly correlated with a shorter duration (≤4 weeks) of symptoms or signs, with complete recovery noticed in 78.6%, versus 25% for patients with a longer symptom duration (P=0.008). Among the patients with partial or no neurological recovery, none exhibited MRI evidence of significant tumor-mass compression of their spinal cord following upfront medical treatment (chemotherapy/chemotherapy plus steroids), and consequently they were not indicated for any further locally directed treatment approaches. The type of upfront treatment, patient age and site of the primary tumor did not show a statistically significant difference when correlated with the neurological outcome (Tables III and IV).

## Discussion

In total, ~5% of all newly diagnosed NB patients will present with varying neurological manifestations associated with cord compression as motor weakness, pain and sensory loss (5), and this will reach up to 19% among children with stage 2/3 disease (3). Among the reviewed patients, 6% had various neurological deficits. The majority of these patients were diagnosed as high risk, disease stage 3 and 4.

The optimal specific therapeutic approach for NB patients with epidural compression was largely debatable. While none of the present study patients who had started on chemotherapy required further neurosurgical intervention or radiotherapy to relieve sequlae of compression, the efficacy of various treatment modalities has been evaluated in numerous previous studies. Historically, adults with varied malignant etiologies and MSCC showed that a combination of surgery followed by radiotherapy was found to be associated with the greatest functional improvement compared with treatment of each individually (6). At level 3 of evidence, children with soft-tissue or bone sarcomas with severe cord compression were shown to have an improved neurological recovery in response to laminectomy compared with chemotherapy or radiotherapy (7). However, laminectomy has been found to be associated with a high rate of spinal instability (8). In addition, previous data has shown that the majority of patients with intraspinal NB leading to neurological impairment should recover regardless of undergoing laminectomy (9). In general, such tumors are responsive to chemotherapy and several studies have documented an equivalent outcome for patients treated with chemotherapy or laminectomy with regards to the associated neurological complications (3,5,9). Thus, first-line chemotherapy has also been recommended for use on a type 3 level of evidence, particularly in extremely young children, in order to decrease the long-term sequelae of laminectomy or spinal radiation. Recovery of neurological functions and regression of the tumor in response to chemotherapy has been reported in children with NB having similar or an improved outcome compared with those who underwent laminectomy or radiation (10). Current recommendations mainly advise an emergency neurosurgical decompression only in cases of neurological progression during chemotherapy (3,11).

The timing of specific therapy initiation has a great effect on determining the neurological outcome. An earlier study demonstrated that early diagnosis may prevent permanent disability in children with malignant cord compression (12). By constrast, it has been reported that a complete recovery of neurological manifestations was possible, despite long time intervals between the occurrence of symptoms and the initiation of treatment, and vice versa (3). In the present study, compression manifestations of <4 weeks duration carried an improved outcome compared with a longer time compression, with complete recovery in 78.6%, versus 25% for patients with a longer symptom duration (P=0.008).

The role of dexamethazone in spinal cord compression has been shown to reduce edema, inhibit inflammatory responses, stabilize vascular membranes and delay the onset of neurological deficit (3,13). Certain studies have shown that there is good evidence that moderate- to high-dose dexamethasone can be recommended for patients with MSCC and significant neurological dysfunction (14). In a randomized comparison, 48 patients assigned to radiotherapy with or without corticosteroids for MSCC had a significantly improved ambulatory outcomes when on the dexamethasone arm, compared with others (P<0.05) (1,15). Additional steroid therapy did not significantly affect the outcome in patients who were already initiated on concomitant chemotherapy or underwent surgical decompression (P=0.37; Table IV), which may be explained by the dominating effect of aggressive chemotherapy that could have overlooked the dehydrating anti-inflammatory actions of the corticosteroids.

Spinal cord compression in NB can be effectively managed with upfront chemotherapy. Initial surgical decompression should be reserved for benign variants only, such as ganglioneuroma. Neurological manifestations of ≤4 weeks duration at presentation are usually reversible.

## Figures and Tables

**Table I:** Characteristic features of the 51 neuroblastoma patients with intraspinal extension.

	Neurological manifestations, n (%)		
	
Feature	Present	Absent	Total
Gender			
Female	17 (50)	8 (47.1)	25 (49.0)
Male	17 (50)	9 (52.9)	26 (51.0)
Age, months (median: 31.8 m)			
≤18	13 (38.2)	10 (58.8)	23 (45.1)
>18	21 (61.8)	7 (41.2)	28 (54.9)
Pathology			
Ganglioneuroma	1 (2.9)	1 (5.9)	2 (3.9)
Ganglioneuroblastoma	4 (11.8)	2 (11.8)	6 (11.8)
Neuroblastoma	29 (85.3)	14 (82.4)	43 (84.3)
Shimada			
Favorable	10 (29.4)	5 (29.4)	15 (29.4)
Unfavorable	18 (52.9)	12 (70.6)	30 (58.8)
Not applicable	6 (17.6)	-	6 (11.8)
NMYC gene status			
Amplified	5 (14.7)	3 (17.6)	8 (15.7)
Non-amplified	20 (58.8)	13 (76.5)	33 (64.7)
Not done	9 (26.5)	1 (5.9)	10 (19.6)
Primary site			
Mediastinal	14 (41.2)	2 (11.8)	16 (31.4)
Pelviabdominal	3 (8.8)	2 (11.8)	5 (9.8)
Retroperitoneal	13 (38.3)	8 (47.1)	21 (41.2)
Suprarenal	4 (11.8)	5 (29.4)	9 (17.6)
Stage			
1	1 (2.9)	-	1 (2.0)
2	2 (5.9)	1 (5.9)	3 (5.9)
3	13 (38.3)	8 (47.1)	21 (41.2)
4	17 (50)	8 (47.1)	25 (49.0)
4S	1 (2.9)	-	1 (2.0)
Risk stratification			
High	22 (64.7)	4 (23.5)	26 (51.0)
Intermediate	10 (29.4)	12 (70.6)	22 (43.1)
Low	2 (5.9)	1 (5.9)	3 (5.9)

**Table II:** Personal and neurological criteria of the 34 neuroblastoma patients with manifest neurological deficits.

			Neurological deficits				
			
Serial	Gender	Age, years	Paresis	Pain	Sphincteric	Duration	Course
1	M	1	Y	-	-	2	P
2	M	1.4	Y	-	-	2	P
3	M	1.4	Y	-	-	2	C
4	F	0.2	Y	-	-	2	P
5	M	3.4	Y	-	-	2	S
6	F	2.9	Y	-	-	1	C
7	M	4.3	Y	-	-	1	P
8	M	8	Y	-	-	2	C
9	F	1	Y	-	-	2	S
10	M	3	Y	-	-	2	P
11	F	1.7	Y	-	-	1	C
12	M	3.6	-	Y	-	2	C
13	F	4.2	Y	Y	-	1	P
14	M	3.2	Y	-	(A)	2	S
15	M	6.6	Y	-	-	1	C
16	F	1.5	Y	-	-	1	C
17	F	2	Y	-	-	2	S
18	F	2.2	Y	-	-	1	C
19	M	4.5	Y	-	(U)	1	C
20	M	13.3	Y	-	-	1	C
21	F	0.6	Y	-	(A), (U)	2	P
22	F	4.4	Y	-	-	2	P
23	M	0.7	Y	-	-	2	C
24	F	1	Y	-	-	1	C
25	F	0.6	Y	-	-	2	P
26	F	1.4	Y	-	-	1	S
27	F	0.5	Y	-	-	1	P
28	M	4	-	Y	-	2	C
29	F	2.8	Y	-	(U)	2	S
30	M	0.9	Y	-	-	2	P
31	F	7	Y	-	-	1	C
32	M	2.7	Y	Y	-	2	S
33	F	3.8	Y	-	-	1	C
34	M	2.6	Y	-	-	1	C

M, male; F, female; Y, yes; (U), urinary; (A), anal; 1, ≤4 weeks; 2, >4 weeks; C, completely recovered; P, partially recovered; S, stationary course.

**Table III:** Duration of neurological manifestations in association with the study parameters of the 34 patients with neurological manifestations.

Study parameter	≤4 weeks	>4 weeks	P-value
Age, months			0.51
≤18	3	10	
>18	11	10	
Stage			0.09
1	1	0	
2	2	0	
3	3	10	
4	8	9	
4S	0	1	
Risk			0.18
Low	2	0	
Intermediate	3	7	
High	9	13	
Upfront treatment			0.34
CTH only	4	11	
CTH+steroids	8	7	
Decompression surgery	2	2	
Neurological outcome			0.008
Complete recovered	11	5	
Partially recovered	2	9	
Stationary	1	6	

CTH, chemotherapy.

**Table IV:** Neurological outcome in association with the study parameters of the 34 patients with neurological manifestations.

	Neurological outcome, recovery			
	
Study parameter	Complete	Partial	No (stationary)	P-value
Age, month				0.12
≤18	4	7	2	
>18	12	4	5	
Stage				0.14
1	1	0	0	
2	2	0	0	
3	3	7	3	
4	10	3	4	
4S	0	1	0	
Risk				0.24
Low	2	0	0	
Intermediate	2	5	3	
High	12	6	4	
Upfront treatment				0.37
CTH only	7	3	5	
CTH + Steroids	6	7	2	
Decompression surgery	3	1	0	
Duration, weeks				0.008
≤4	11	2	1	
>4	5	9	6	

CTH, chemotherapy.
